# Cardiac Septal Occluder for Refractory Anastamotic Leak

**DOI:** 10.1093/jcag/gwac019

**Published:** 2022-05-31

**Authors:** Marcel Tomaszewski, Cameron McAlister, Janarthanan Sathananthan, Fergal Donnellan

**Affiliations:** Department of Medicine, University of British Columbia, Vancouver, Canada; Division of Gastroenterology, Vancouver General Hospital, Vancouver, Canada; Division of Cardiology, Vancouver General Hospital, Vancouver, Canada; Division of Cardiology, Vancouver General Hospital, Vancouver, Canada; Division of Gastroenterology, Vancouver General Hospital, Vancouver, Canada

A 69-year-old lady underwent a total gastrectomy with roux-en-Y reconstruction for diffuse type gastric cancer.

**Figure 1.**
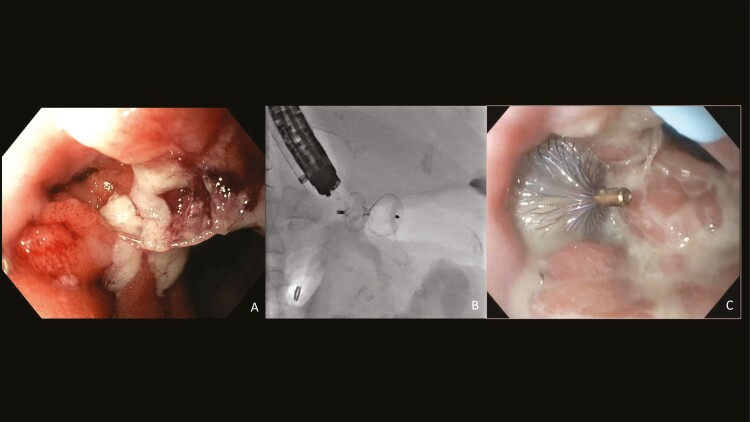
(A) A 5-mm fistula tract was identified at the esophagojejunal anastomosis. (B and C) A 9-mm waisted Amplatzer septal occluder was deployed across the fistula tract beside the endoscope under fluoroscopic (B) and endoscopic guidance (C).

Increased output from the abdominal incision site occurred with oral fluid intake. An enterocutaneous fistula was suspected. Upper endoscopy demonstrated a fistula of 5 mm in diameter at the esophagojejunal anastomosis (A). Despite placement of a fully covered esophageal stent with over the scope clip fixation, oral fluid continued to drain via the abdominal wound. CT scan demonstrated a large collection in the left upper quadrant communicating with the abdominal wound and the fistula at the esophagojejunal anstamosis (Figure 1).

Two months after the initial surgery, the esophageal stent and over the scope clip were removed. A pediatric gastroscope was passed through the esophagojejunal anastamotic fistula into the collection. Under endoscopic and fluoroscopic guidance, a Savary guidewire was passed into the collection through the fistula tract. A 7 french Amplatzer sheath was then passed over the wire, beside the endoscope, into the collection. A 9-mm Amplatzer septal occluder was deployed across this fistula tract, with one disk in the collection and the other disk in the gut (B, C). The collection was managed with a percutaneous drain for 2 weeks. At 5 weeks post-fistula closure, drainage from the abdominal wound had stopped.

Gastrointestinal fistulae are notoriously challenging to close with the many endoscopic closure options available demonstrating suboptimal clinical results (1). The Amplatzer septal occluder is predominantly used in the management of cardiac septal defects but can be used to plug refractory gastrointestinal fistulae (2).
